# Association of random glucose to albumin ratio with post-contrast acute kidney injury and clinical outcomes in patients with ST-elevation myocardial infarction

**DOI:** 10.3389/fendo.2024.1390868

**Published:** 2024-06-18

**Authors:** Ping Lai, Xiaoyan Gu, Xuhui Lin, Yu He, Yining Dai, Chongyang Duan, Yuanhui Liu, Wenfei He

**Affiliations:** ^1^ Department of Cardiology, First Affiliated Hospital of Gannan Medical University, Key Laboratory of Prevention and Treatment of Cardiovascular and Cerebrovascular Diseases, Ministry of Education, Gannan Medical University, Ganzhou, China; ^2^ Department of Endocrinology, The Fifth Affiliated Hospital of Guangzhou Medical University, Guangzhou, China; ^3^ Department of Cardiology, Guangdong Cardiovascular Institute, Guangdong Provincial People’s Hospital (Guangdong Academy of Medical Sciences), Southern Medical University, Guangzhou, China; ^4^ Guangdong Provincial Key Laboratory of Coronary Heart Disease Prevention, Guangdong Provincial People’s Hospital, Guangdong Academy of Medical Sciences, Guangzhou, China; ^5^ Department of Biostatistics, School of Public Health, Southern Medical University, Guangzhou, China; ^6^ Department of Cardiology, Guangdong Provincial People’s Hospital’s Nanhai Hospital, The Second People’s Hospital of Nanhai District, Foshan, China

**Keywords:** random glucose, albumin, post-contrast acute kidney injury, ST-segment elevation myocardial infarction, percutaneous coronary intervention

## Abstract

**Purpose:**

Both glucose and albumin are associated with chronic inflammation, which plays a vital role in post-contrast acute kidney injury (PC-AKI). To explore the relationship between random glucose to albumin ratio (RAR) and the incidence of PC-AKI after percutaneous coronary intervention (PCI) in patients with ST-elevation myocardial infarction (STEMI).

**Patients and methods:**

STEMI patients who underwent PCI were consecutively enrolled from January, 01, 2010 to February, 28, 2020. All patients were categorized into T1, T2, and T3 groups, respectively, based on RAR value (RAR < 3.377; 3.377 ≤ RAR ≤ 4.579; RAR > 4.579). The primary outcome was the incidence of PC-AKI, and the incidence of major adverse clinical events (MACE) was the second endpoint. The association between RAR and PC-AKI was assessed by multivariable logistic regression analysis.

**Results:**

A total of 2,924 patients with STEMI undergoing PCI were finally included. The incidence of PC-AKI increased with the increasing tertile of RAR (3.2% vs 4.8% vs 10.6%, P<0.001). Multivariable regression analysis demonstrated that RAR (as a continuous variable) was associated with the incidence of PC-AKI (adjusted odds ratio (OR) =1.10, 95% confidence interval (CI) =1.04 - 1.16, P<0.001) and in-hospital MACE (OR=1.07, 95% CI=1.02 - 1.14, P=0.012); RAR, as a categorical variable, was significantly associated with PC-AKI (T3 vs. T1, OR=1.70, 95% CI=1.08 - 2.67, P=0.021) and in-hospital MACE (T3 vs. T1, OR=1.63, 95% CI=1.02 - 2.60, P=0.041) in multivariable regression analyses. Receiver operating characteristic curve analysis showed that RAR exhibited a predictive value for PC-AKI (area under the curve (AUC)=0.666, 95% CI=0.625 - 0.708), and in-hospital MACE (AUC= 0.662, 95% CI =0.619 - 0.706).

**Conclusions:**

The high value of RAR was significantly associated with the increasing risk of PC-AKI and in-hospital MACE after PCI in STEMI patients, and RAR offers a good predictive value for those outcomes.

## Introduction

Post-contrast acute kidney injury (PC-AKI) is one of the most common comorbidities following percutaneous coronary intervention (PCI), which is significantly higher in patients with ST-segment elevated myocardial infarction (STEMI) than other patients (1, 2). Patients with PC-AKI have higher mortality, and longer hospitalization than patients without PC-AKI (3). However, there is no effective treatment for PC-AKI to date (4), identifying patients at high-risk of PC-AKI and implementing timely preventative measures are critical in avoiding PC-AKI.

In clinical, the constantly updated risk score is used to predict PC-AKI after PCI (5, 6). However, most risk factors for PC-AKI included in the risk score were largely unchangeable and irreversible, making them unsuitable for primary PCI since most of those parameters are not readily available. Investigating some novel and potentially modifiable predictors may help to minimize PC-AKI incidence. Recent studies found non-diabetic patients with elevated pre-procedural random glucose have a higher risk of PC-AKI (7, 8). Both fasting glucose and random glucose can predict in-hospital events in STEMI patients, but random glucose is more convenient in real-time and easier to obtain (9). Low serum albumin has been demonstrated as a potential prognostic marker and predictor of various inflammatory diseases and PC-AKI (10–13). Furthermore, the ratio of fibrinogen to albumin in the blood was successfully used to predict PC-AKI (14). Glucose and albumin have both been adapted as critical parameters for monitoring the dynamic changes in renal function (15, 16). In terms of predicting renal function, a paradox exists between glucose and albumin, with higher glucose and lower albumin indicating worse renal function (15, 16). Therefore, we hypothesize that the random glucose to albumin ratio (RAR) could be a novel predictor for PC-AKI in patients with STEMI which may be helpful for the prevention of PC-AKI. The primary objective of this study was to assess the association between RAR and PC-AKI and other outcomes among patients with STEMI underwent PCI.

## Patients and method

### Study design and patients

The present study on the predictive value of RAR for PC-AKI was conducted at the Guangdong Provincial People’s Hospital between January 2010 and February 2020. Patients with STEMI undergoing PCI were consecutively enrolled in this observational cohort study. STEMI was diagnosed using the latest criteria from the 2017 ESC Guidelines (1). The following were the exclusion criteria: (1) Patients on renal replacement therapy; (2) Contrast agent allergies; (3) Patients without receiving the percutaneous coronary intervention; (4) A history of severe chronic inflammatory disease or a malignant tumor, and steroidal agents’ treatment recently; (5) Undergoing coronary artery bypass grafting; (6) Random glucose or albumin values were missing. The ethics committee of Guangdong Provincial People’s Hospital approved the study, and all patients signed a written informed consent before the procedure.

### Study protocol

The medical information recording systems were used to collect patient demographic and clinical characteristics such as age, sex, smoking status, medical history, laboratory indices, echocardiography, angiographic variables, and medication used during hospitalization. All laboratory examinations were systematically and preoperatively performed in Guangdong Provincial People’s Hospital. RAR was calculated using the random glucose/albumin formula. Random glucose was measured by analyzed biochemically using blood collected before PCI, and the albumin value was measured within 6 hour after PCI. SCr levels were measured before and after PCI for a period of 2–3 days. We evaluated the estimated glomerular filtration rate using the modified Modification of Diet in Renal Disease equation for Chinese patients (17).

PCI was performed using standard guide catheters, guidewires, balloon catheters, and stents via the femoral or radial approach, in accordance with standard clinical practice. All patients received nonionic, low-osmolarity contrast agents. In addition, patients received 0.9% saline (1 ml/kg/h) during the procedure and maintained for 6–12 hours afterward.

### Primary and second endpoints

The primary endpoint was PC-AKI development, defined as an increase in serum creatinine (SCr) of more than 44.2 μmol/L (0.5 mg/dL) from baseline in the initial 48 to 72 hours after contrast exposure (4). The second endpoint was the occurrence of major adverse clinical events (MACE), which included all-cause mortality, recurrent myocardial infarction, stroke, or target vessel revascularization during hospitalization. The other definition of PC-AKI, as an increase in SCr of more than 26.4 μmol/L (0.3 mg/dL) from baseline in the initial 48 to 72 hours after contrast exposure, was also reported (4, 18).

### Statistical analysis

Baseline characteristics of participants who were divided into three groups according to the tertile of RAR: T1 (n=974, RAR < 3.377), T2 (n=975, 3.377 ≤ RAR ≤ 4.579), and T3 (n=975, RAR > 4.579) were compared. The mean and standard deviation (SD) of normally distributed continuous variables were calculated and analyzed using Student’s *t*-tests. The Wilcoxon ranksum test was used to analyze nonnormally distributed variables that were expressed as medians or quartile. Categorical variables were represented as percentages and analyzed using the chi-square or Fisher exact test.

For risk factors of PC-AKI and in-hospital MACE, odds ratios (OR) with 95 percent confidence intervals (CI) were calculated using univariable and multivariable logistic regression analyses. Variables that were statistically significant in the univariate analysis and those known to be related to infection (according to previous studies) were adjusted in multivariable logistic regression analyses. Risk factors (age, gender, heart failure, smoke, hypertension, chronic obstructive pulmonary disease, previous myocardial infarction, prior PCI, previous stroke, anemia, estimated glomerular filtration rate) and variables (aspirin, GPIIb/IIIa inhibitor, multi-vessel stenosis, femoral access) which were validated to be meaningful in medical practice, were included in the multiple logistic regression analysis. A cubic spine model (adjusted for age and gender) was also performed to judge the effect of RAR on PC-AKI and MACE. Receiver operating characteristic (ROC) curve analysis was used to evaluate the value of RAR levels for predicting PC-AKI and MACE, and the area under the ROC curve (AUC) was subsequently calculated. The AUC value can be used to evaluate the efficacy of the predictor (AUC <0.6, poor discrimination; 0.6 – 0.75, good discrimination; >0.75, excellent discrimination) (19). The optimal cutoff value was determined by using the Youden index.

SAS version 9.4 (SAS Institute, Cary, NC, USA) was used for all statistical analyses. All probability values were two-tailed, and statistical significance was defined as *P* value less than 0.05.

## Results

### Baseline characteristics of all groups

**Figure 1:**
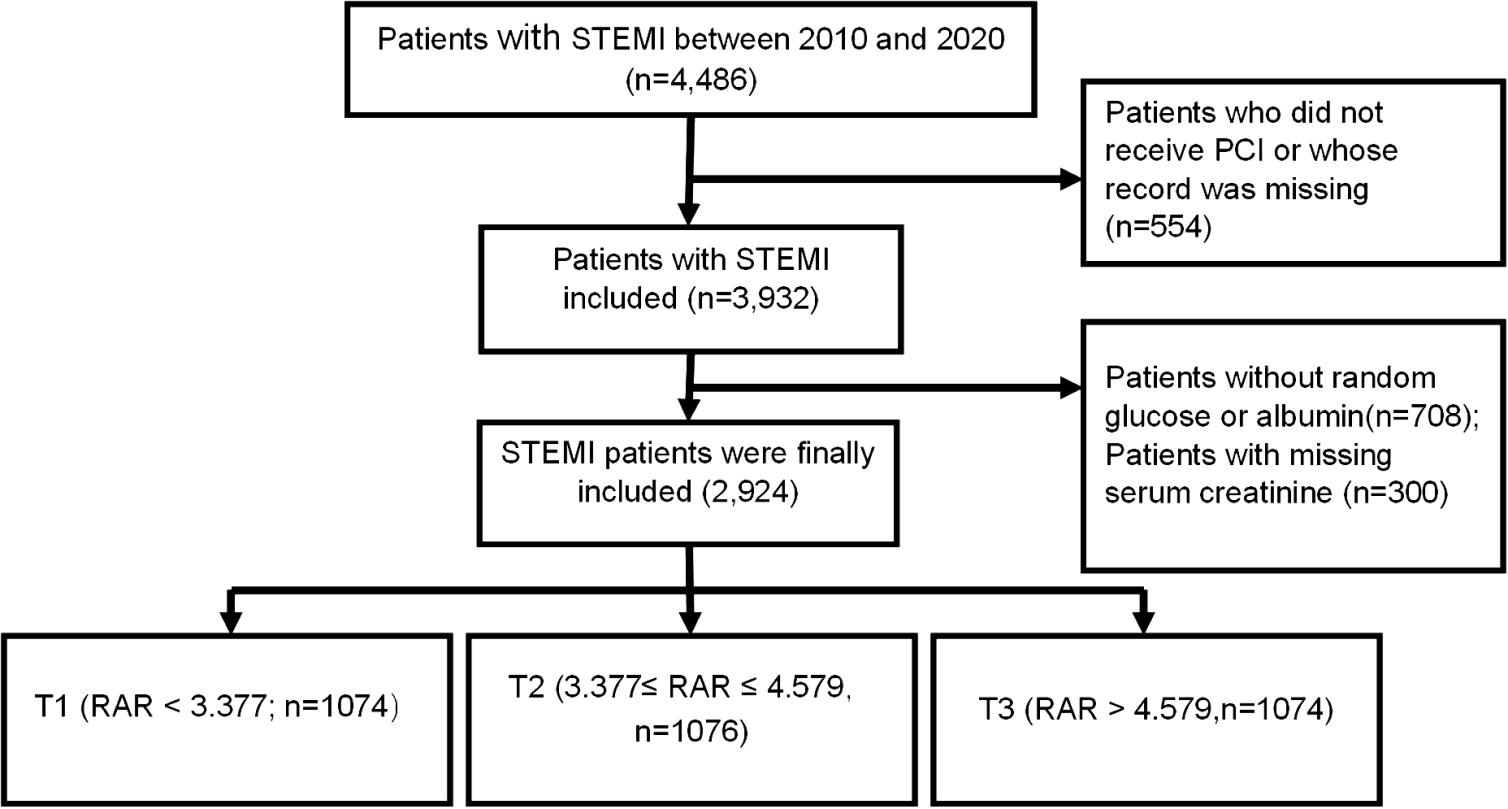
Flowchart of participants.

A total of 2,924 STEMI patients undergoing PCI were finally enrolled (**Figure 1**). The average age was 62.30 ± 12.00 years, 43.39% of the total patients were more than 65 years, and 2,411 (82.46%) were male. Patients were divided into the three groups based on the tertiles of the RAR values: T1(n=974): RAR < 3.377; T2 (n=975): 3.377 ≤ RAR ≤ 4.579; T3 (n=975):RAR > 4.579. Random blood glucose was significantly higher in the T3 group, while, albumin was significantly lower. The percentages of hypertension, heart failure, diabetes, and smoker were higher in the T3 group. Patients with higher RAR received more insulin, clopidogrel, angiotensin-converting enzyme inhibitor/angiotensin receptor blocker, aspirin, glycoprotein IIb/IIIa receptor inhibitor, beta-blockers, and calcium channel blocker drugs and more stents; and were more likely to have multi-vessel stenosis than those with lower RAR (**Table 1**).

**Table 1:** Baseline characteristics of patients enrolled in this study were stratified by RAR’s tertile.

Variables	All patients (n=2924)	T1 (n=974) RAR ≤ 3.377	T2 (n=975) 3.377<RAR<4.579	T3 (n=975) RAR≥4.579	P value
Demographics and clinical characters					
Age, years	62.30 ± 12.00	58.96 ± 12.43	63.21 ± 12.15	64.72 ± 11.40	<.001
Male, n (%)	2411 (82.46)	865 (88.8)	816 (83.7)	730 (74.9)	<.001
SBP (mmHg)	121.96 ± 21.82	123.73 ± 20.35	121.70 ± 22.08	120.48 ± 23.03	<.001
DBP (mmHg)	74.16 ± 13.47	75.80 ± 13.45	74.12 ± 13.47	72.56 ± 13.49	<.001
NYHA classification, n (%)					
I	2031 (69.50)	771 (79.2)	684 (70.2)	576 (59.1)	<.001
II	608 (20.79)	160 (16.4)	209 (21.5)	239 (24.5)	.
III	152 (5.20)	27 (2.8)	44 (4.5)	81 (8.3)	.
IV	132 (4.51)	16 (1.6)	37 (3.8)	79 (8.1)	.
Comorbidities, n (%)					
Smoke	1221 (41.76)	490 (50.4)	392 (40.2)	339 (34.8)	<.001
Hypertension	1506 (51.50)	442 (45.4)	503 (51.6)	561 (57.5)	<.001
Diabetes	849 (29.04)	69 (7.1)	155 (15.9)	625 (64.1)	<.001
COPD	66 (2.26)	19 (2.0)	25 (2.6)	22 (2.3)	0.660
Previous MI	604 (20.66)	233 (23.9)	162 (16.6)	209 (21.4)	<.001
Prior-PCI	369 (12.62)	109 (11.2)	130 (13.3)	130 (13.3)	0.259
Previous Stroke	215 (7.35)	48(4.9)	63(6.5)	104(10.7)	<.001
Previous atrial fibrillation	98 (3.35)	28 (2.9)	32 (3.3)	38 (3.9)	0.450
Laboratory measurements					
Random blood glucose, mmol/L	8.70 ± 2.10	5.83 ± 0.80	7.53 ± 0.99	12.74 ± 4.53	<.001
Albumin, mmol/L	34.88 ± 4.06	36.97 ± 3.70	34.68 ± 3.88	32.98 ± 4.59	<.001
LVEF (%)	51.08 ± 11.73	53.00 ± 11.10	51.42 ± 11.62	48.83 ± 12.46	<.001
Hemoglobin, g/L	134.37 ± 18.94	138.72 ± 16.62	134.13 ± 18.24	130.26 ± 21.95	<.001
Anemia, n (%)	924 (31.6)	230 (23.7)	319 (32.8)	375 (38.5)	<.001
Baseline creatinine, mg/dL	0.98 (0.83~1.20)	0.94 (0.80~1.10)	0.97 (0.83~1.14)	1.04 (0.85~1.37)	<.001
eGFR, ml/min	83.72 (64.95~102.38)	90.15 (73.32~108.42)	85.17 (68.85~102.21)	75.85 (52.68~96.52)	<.001
HbA1c, %	6.4 (5.5~9.20)	5.80 (5.50~6.10)	6.00 (5.60~6.40)	7.40 (6.30~9.20)	<.001
Total Cholesterol, mmol/L	4.89 ± 1.23	5.04 ± 1.21	4.84 ± 1.16	4.78 ± 1.33	<.001
LDL-C, mmol/L	3.20 ± 1.01	3.35 ± 0.98	3.16 ± 0.98	3.10 ± 1.06	<.001
HDL-C, mmol/L	1.00 ± 0.26	1.01 ± 0.27	1.02 ± 0.25	0.96 ± 0.26	<.001
Triglyceride, mmol/L	1.40 (1.02~1.92)	1.45 (1.04~2.02)	1.28 (0.97~1.78)	1.46 (1.06~1.97)	<.001
Hypernatremia, n (%)	31 (1.1)	7 (0.7)	4 (0.4)	20 (2.1)	<.001
Serum sodium	137.57 ± 4.28	138.24 ± 3.62	137.49 ± 5.28	136.98 ± 3.93	<.001
Medication use during hospitalization, n (%)					
Aspirin	2888 (98.77)	965 (99.1)	968 (99.3)	955 (97.9)	0.016
Clopidogrel	2595 (88.75)	834 (85.6)	870 (89.4)	891 (91.5)	<.001
Statins	2861 (97.85)	954 (97.9)	957 (98.3)	950 (97.5)	0.538
Metformin	204 (6.78)	17 (1.7)	39 (4.0)	148 (15.3)	<.001
Betablocker	2392 (81.81)	815 (83.7)	794 (81.5)	783 (80.4)	0.161
Insulin	472 (16.14)	14 (1.4)	57 (5.9)	401 (41.2)	<.001
ACEI/ARB	2370 (81.05)	812 (83.4)	792 (81.2)	766 (78.6)	0.025
Calcium channel blocker	282 (9.64)	92 (9.5)	81 (8.3)	109 (11.2)	0.094
Procedural characteristics					
Multi-vessel stenosis, n (%)	2109 (72.13)	659 (67.7)	712 (73.0)	738 (75.7)	0.001
Number of stents	1.00 (1.00~2.00)	1.00 (1.00~2.00)	1.00 (1.00~2.00)	1.00 (1.00~2.00)	0.017
Length of stent (mm)	31.00 (21.67~49.33)	30.00 (21.00~47.00)	30.00 (21.00~50.00)	33.00 (23.00~51.00)	0.008
Contrast volume (ml)	100.00 (100.00~150.00)	100.00(100.00~150.00)	100.00(100.00~150.00)	100.00(100.00~150.00)	0.072
Length of hospital stay, days	6.67(5.00~11.00)	6.00 (5.00~8.00)	7.00 (5.00~9.00)	7.00 (6.00~11.00)	<.001

SBP, systolic blood pressure; DBP, Diastolic blood pressure; COPD, chronic obstructive pulmonary disease; MI, myocardial infarction; PCI, percutaneous coronary intervention; LVEF, left ventricular ejection fraction; WBC, white blood cell; eGFR, estimated glomerular filtration rate; HbA1c, glycosylated hemoglobin; LDL-C, low-density lipoprotein cholesterol; HDL-C, high-density lipoprotein cholesterol; ACEI/ARB, angiotensin converting enzyme inhibitor/angiotensin receptor blocker.

### RAR correlates with PC-AKI and in-hospital MACE

**Figure 2:**
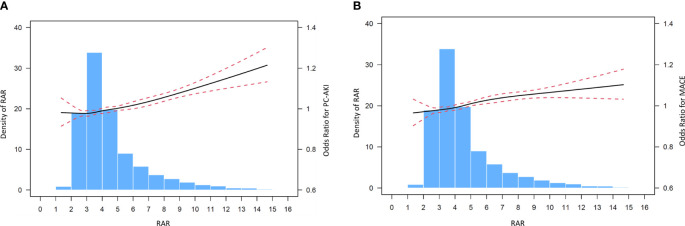
**(A)** Cubic spine models for the association between RAR and PC-AKI. **(B)** Cubic spine models for the association between RAR and in hospital MACE.

The incidence of PC-AKI significantly rose with increasing of RAR. Only 3.2% of the patients in T1 developed PC-AKI, while it was as high as 10.6% in the T3 group. Cubic spline models demonstrated no significant non-linear relationship between RAR and MACE (**Figures 2A**, **B**). The incidence of each endpoint included in MACE was listed in **Supplementary Table 1**.

Multivariable regression analyses demonstrated that RAR, as a continuous variable, was associated with the incidence of PC-AKI (OR=1.10, 95% CI=1.04 - 1.16, *P<0.001*) and in-hospital MACE (OR=1.07, 95% CI=1.02 - 1.14, *P*=0.012) in patients with STEMI undergoing PCI (**Table 2**). Furthermore, RAR, as a continuous variable, was also associated with the incidence of other definition of PC-AKI (OR=1.09, 95% CI=1.04 - 1.14, *P<0.001*) (**Supplementary Table 2**). Meanwhile, RAR, as a categorical variable, was related to PC-AKI (T3 *vs.* T1, OR=1.70, 95% CI=1.08 - 2.67, *P*=0.021), other definition of PC-AKI (T3 *vs.* T1, OR=1.60, 95% CI=1.14 - 2.26, *P*=0.007), and in-hospital MACE (T3 *vs.* T1, OR=1.63, 95% CI=1.02–2.60, *P*=0.041) in multivariable regression analyses(**Table 3** and **Supplementary Table 3**). Furthermore, the result remained that RAR (as a continuous or categorical variable) was related to PC-AKI and in hospital MACE after adjusting the history of diabetes, and proved that RAR is associated with clinical outcomes independent of history of diabetes (**Supplementary Table 4**). Another multivariable model also demonstrated similar results (**Supplementary Table 5**).

**Table 2:** Multivariable logistic regression analysis for the RAR as continuous variable.

Variables	PC-AKI			In hospital MACE		
	OR value	95% CI	P value	OR value	95% CI	P value
RAR, per1-unit increase	1.10	1.04~1.16	0.000	1.07	1.02~1.14	0.012
Age	1.04	1.02~1.06	0.000	1.02	1.00~1.03	0.048
Female	0.56	0.36~0.88	0.011	1.08	0.69~1.69	0.750
Heart failure	2.09	1.49~2.94	0.000	2.00	1.39~2.87	0.000
Smoke	0.92	0.64~1.33	0.652	1.10	0.75~1.62	0.626
Hypertension	0.99	0.70~1.40	0.946	1.07	0.74~1.54	0.724
COPD	0.64	0.27~1.52	0.315	1.10	0.46~2.63	0.821
Previous MI	0.48	0.30~0.79	0.004	0.70	0.43~1.15	0.161
Prior PCI	0.86	0.52~1.41	0.555	0.74	0.42~1.28	0.281
Previous Stroke	1.77	1.13~2.78	0.013	1.27	0.76~2.13	0.356
Anemia	1.20	0.85~1.69	0.294	0.99	0.68~1.42	0.940
eGFR	0.98	0.97~0.99	0.000	0.98	0.98~0.99	0.000
Aspirin	1.13	0.30~4.20	0.860	0.58	0.18~1.88	0.366
GP IIb/IIIa inhibitor	1.04	0.74~1.47	0.806	1.65	1.16~2.35	0.005
Multi-vessel stenosis	1.14	0.80~1.63	0.476	1.66	1.10~2.50	0.015
Femoral access	1.48	1.02~2.16	0.038	2.13	1.47~3.10	0.000

When the LVEF was added in the multivariable model, the result showed that RAR was related to PC-AKI (OR=1.084, 95%CI:1.027–1.144, P=0.004) and in hospital MACE (OR=1.067, 95% CI: 1.006–1.131, P=0.031); and when the diabetes was added in the multivariable model, the result showed that RAR was related to PC-AKI (OR=1.074, 95% CI:1.011–1.141, P=0.021) and in hospital MACE (OR=1.088, 95% CI: 1.019–1.161, P=0.011).

RAR, random glucose to albumin ratio; COPD, chronic obstructive pulmonary disease; MI, myocardial infarction; PCI, percutaneous coronary intervention; eGFR, estimated glomerular filtration rate; GP IIb/IIIa inhibitor, Glycoprotein IIb/IIIa inhibitor.

**Table 3:** Multivariable logistic regression analysis for the RAR as categorical variable.

Variables	PC-AKI			In hospital MACE		
	OR value	95% CI	P value	OR value	95% CI	P value
T1		Reference			Reference	
T2	0.96	0.59~1.56	0.855	0.92	0.55~1.53	0.754
T3	1.70	1.08~2.67	0.021	1.63	1.02~2.60	0.041
Age	1.04	1.02~1.06	0.000	1.02	1.00~1.03	0.079
Female	0.58	0.38~0.91	0.018	1.10	0.70~1.71	0.688
Heart failure	2.12	1.51~2.98	0.000	2.01	1.40~2.88	0.000
Smoke	0.93	0.64~1.35	0.704	1.11	0.75~1.64	0.596
Hypertension	0.99	0.70~1.40	0.951	1.07	0.74~1.54	0.725
COPD	0.64	0.27~1.53	0.320	1.13	0.48~2.69	0.780
Previous MI	0.46	0.28~0.76	0.002	0.68	0.41~1.11	0.123
Prior PCI	0.85	0.52~1.40	0.527	0.73	0.42~1.28	0.272
Previous Stroke	1.77	1.13~2.77	0.013	1.27	0.76~2.11	0.368
Anemia	1.20	0.85~1.69	0.296	0.98	0.68~1.42	0.922
eGFR	0.98	0.97~0.99	0.000	0.98	0.98~0.99	0.000
Aspirin	1.09	0.30~3.94	0.895	0.59	0.19~1.87	0.371
GP IIb/IIIa inhibitor	1.05	0.75~1.48	0.777	1.66	1.17~2.36	0.005
Multi-vessel stenosis	1.12	0.79~1.60	0.526	1.64	1.09~2.46	0.018
Femoral access	1.46	1.01~2.12	0.046	2.09	1.44~3.04	0.000

COPD, chronic obstructive pulmonary disease; MI, myocardial infarction; PCI, percutaneous coronary intervention; eGFR, estimated glomerular filtration rate; GP IIb/IIIa inhibitor, Glycoprotein IIb/IIIa inhibitor.

### Predictive value of RAR for PC-AKI and in-hospital MACE

**Figure 3:**
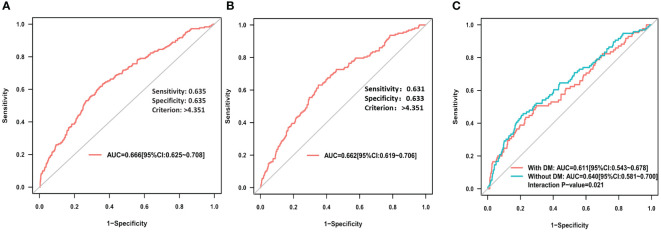
**(A)** ROC curve analysis of RAR for PC-AKI; **(B)** ROC curve analysis of RAR for in hospital MACE; **(C)** ROC curve analysis of RAR for PC-AKI among STEMI patients with or without DM.

ROC curve analysis showed that RAR exhibited a good predictive value for PC-AKI (area under the curve (AUC) = 0.666, 95% CI=0.625 - 0.708), and the optimal cutoff point of RAR was 4.351, with a sensitivity of 63.5% and specificity of 63.5% (**Figure 3A**). Additionally, RAR was demonstrated a similar predictive value for PC-AKI (AUC= 0.633, 95% CI=0.601 - 0.665) based on other definition (**Supplementary Figure 1**). RAR also revealed a predictive value for in-hospital MACE (AUC= 0.662, 95% CI =0.619 - 0.706) (**Figure 3B**).

### Subgroup analysis

The AUC of RAR for predicting PC-AKI in the no diabetes subgroup was significantly higher compared to that in the diabetes subgroup (AUC: 0.640 *vs* 0.611, *P*=0.021) (**Figure 3C**), while, there is not significantly different in subgroup of gender (AUC: 0.659 *vs* 0.725, *P*=0.329) (**Supplementary Figure 2**). However, except for age (p for interaction: <0.001), subgroup analyses of diabetes (p for interaction: 0.554), or gender (p for interaction: 0.533) or hypertension (p for interaction: 0.409) did not identify any significant difference in PC-AKI (**Supplementary Table 6**). All subgroup analyses did not identify any significant difference in MACE (**Supplementary Table 6**).

## Discussion

To the best of our knowledge, current study was the first to evaluate the relationship between RAR and PC-AKI in patient with STEMI undergoing PCI. Especially, most previous discovered risk factors or predictors are unsuitable for primary PCI since most of those parameters are not readily available (20). Our results found that RAR was an independent predictor for PC-AKI and MACE in patients with STEMI undergoing PCI, and exhibited (more than 60%) good predictive value for PC-AKI in the subgroup of patients without diabetes.

Systemic inflammation is closely related to the development of PC-AKI, and systemic inflammatory indexes like systemic immune-inflammation index (SII) have been used to effectively predict PC-AKI in STEMI patients after PCI (21). However, the SII index lacks specificity in predicting PC-AKI since it was also elevated in various cancer patients (22). Patients with high glucose are prone to chronic inflammation (23). High glucose level causes high expression of pro-inflammatory genes in macrophages in non-diabetes patients (24). Cheuk-Kin Kwan et, al found that high glucose level stimulates inflammation and weakens the pro-resolving response at the cellular level (25). More importantly, study confirmed that acute hyperglycemia could induce renal tubular injury (26).

It is widely acknowledged that diabetes, elevated fasting glucose, and impaired glucose tolerance are vital risk factors for PC-AKI (3), while, glucose tolerance testing and HbA1c are difficult to obtain in patients without diagnosed diabetes or in urgent events like STEMI. Random glucose testing is an optimal option in this situation. In clinical trials, researchers discovered that high random blood glucose significantly increased the risk of PC-AKI after PCI in patients with acute coronary syndrome who did not have diabetes (8). Previous study including 13,3792 non-diabetes patients concluded that an increased random glucose value is a risk factor for diabetes (27) and could predict acceptable overall glycemic control in non-insulin-dependent diabetic patients (28). Qurratul Ain et al. further confirmed that random plasma glucose could effectively reflect glycemic control in adults with type 2 diabetes mellitus (29). A rapid and systemic assessment of glucose metabolism in STEMI patients before coronary radiography and PCI is impossible, while random glucose could turn out to be a reliable option for STEMI patients with normal or unknown abnormal glucose level. Although an increase in random glucose levels was related to PC-AKI, its prognostic value was limited due to its values being easily affected by food intake.

Albumin is an important nutrition subject, and concentration of serum albumin is determined by the absolute rate of albumin synthesis, the fractional catabolic rate, the distribution of albumin between the vascular and extravascular compartments, and exogenous albumin loss (30). The combined effects of inflammation and inadequate protein, and caloric intake induce hypoalbuminemia in patients with chronic diseases such as chronic renal failure (31) and cirrhosis (32). A previous study indicates a negative correlation between albumin and C-response protein levels, as well as between albumin and white blood cell levels (33). Investigators found that inflammation and reduced albumin synthesis are linked to a stable declined of serum albumin in hemodialysis patients (30). Except for low albumin closely associated with inflammation, hypoalbuminemia predicted the risk of AKI in in-hospital patients (34) and non-cardiac surgery (35). Meta-analysis further confirmed that hypoalbuminemia is positively correlated with the risk of AKI (13). However, low protein uptake in renal dysfunction could be the root of low serum albumin.

Ongoing inflammation significantly increased the incidence of PC-AKI in those patients undergoing contrast-enhanced CT (36) and the close relationship between inflammation and PC-AKI has already been widely acknowledged (37–39). As discussed above, random glucose and serum albumin are positively and negatively associated with the pathophysiological process of inflammation, respectively. And a combination of random glucose and serum albumin might be more reliable than random glucose and serum albumin alone in predicting PC-AKI as both random glucose is influenced by daily diet. Notably, the current study provides a reliable foundation for finding potential PC-AKI patients and MACE by RAR, which possesses a higher predictive value in PC-AKI assessments. Considering the analysis conducted, and the metabolic pathways of glucose and albumin are intricately linked to inflammatory processes, which are pivotal in the pathogenesis of PC-AKI. Hence, it is logical to posit that the ratio of glucose to albumin correlates with the incidence of PC-AKI.

Previous researches reported that around 70% of the STEMI patients were male, and 81.3% of them received PCI (40, 41),which is similar to the present study (82.46% male patients receiving PCI). In another more than ten years long-term following up study on STEMI patients, only 17% of them were female (254 in 1498) and there were no differences in the combined patient-oriented endpoint between women and men (42). In addition, although subgroup analyses did not identify any significant difference in PC-AKI after adjustment of other potential factors, the RAR has a little better performance in non-diabetic patients. Several potential reasons should be considered. Firstly, prediabetic patients would be included in the non-diabetic group. Secondly, the random glucose was not affected by anti-diabetic agents in the non-diabetic patients. Thirdly, the operator may pay more attention to those patients with diabetes during the operation since it is well known that diabetes is a main risk factor for PC-AKI. However, the relationship between RAR and PC-AKI in the non-diabetic patients should be evaluated in the future researches with large sample size.

In clinical practice, patients at high risk of PC-AKI based on RAR should be received implementing timely preventative measures avoiding PC-AKI. These patients should be received the standard hydration and monitoring the level of serum creatinine, and avoiding or correcting the low serum albumin. However, further randomized controlled trials with large sample sizes are warranted to validate and optimize the clinical application of RAR on preventing the development of PC-AKI.

## Limitation

Firstly, although we have performed the multivariable analysis (including RAR as continuous or categorical variables) and cubic spine model to test the robustness of RAR predictions for PC-AKI, potential bias was inevitable as an observational study, such as metabolic control, the inflammatory state of the population, and nutritional status. Secondly, due to the different diagnostic criteria of PC-AKI (3), the results may not be repeatable when other PC-AKI definition was used. Lastly, only STEMI patients were included in this study, the result may vary for other types of acute coronary syndrome patients.

## Conclusion

A high value of RAR was associated with an increased risk of PC-AKI and in-hospital MACE in patients with STEMI undergoing PCI, with RAR showing good predictive value for these outcomes.

## Data availability statement

The original contributions presented in the study are included in the article/**Supplementary Material**. Further inquiries can be directed to the corresponding author.

## Ethics statement

The studies involving humans were approved by the ethics committee of Guangdong Provincial People’s Hospital. The studies were conducted in accordance with the local legislation and institutional requirements. The participants provided their written informed consent to participate in this study. The animal study was approved by the ethics committee of Guangdong Provincial People’s Hospital. The study was conducted in accordance with the local legislation and institutional requirements.

## Author contributions

PL: Data curation, Investigation, Writing – original draft, Writing – review & editing. XG: Data curation, Methodology, Writing – original draft. XL: Data curation, Methodology, Writing – original draft. YH: Data curation, Methodology, Writing – original draft. YD: Data curation, Formal analysis, Writing – original draft. CD: Data curation, Investigation, Methodology, Writing – original draft. YL: Investigation, Writing – original draft. WH: Conceptualization, Writing – original draft, Writing – review & editing.
