# Supplementary material for: Association of random glucose to albumin ratio with post-contrast acute kidney injury and clinical outcomes in patients with ST-elevation myocardial infarction
Source: Front Endocrinol (Lausanne). 2024 Jun 18;15:1390868. doi: 10.3389/fendo.2024.1390868 (PMC11217170; doi:10.3389/fendo.2024.1390868)

**Additional file material**

**Association of random glucose to albumin ratio with post-contrast acute kidney injury and clinical outcomes in patients with ST-elevation myocardial infarction**

**Additional file 1: Table S1-6, Figure S1-2**

**Supplementary Table 1:** The occurrence of MACE in each group.

| **MACE** | **T1** | **T2** | **T3** | **P value** |
| --- | --- | --- | --- | --- |
| Stroke | 7 (0.7 %) | 5 (0.5 %) | 21 (2.2 %) | <.001 |
| Target vessel revascularization | 6 (0.6 %) | 9 (0.9 %) | 6 (0.6 %) | 0.652 |
| Recurrent myocardial infarction | 5 (0.5 %) | 6 (0.6 %) | 6 (0.6 %) | 0.943 |
| All-cause mortality | 16 (1.6 %) | 25 (2.6 %) | 70 (7.2 %) | <.001 |

Abbreviation: MACE: major adverse clinical events.

**Supplementary Table 2.** Multivariable logistic regression analysis for the RAR as continuous variable in other definition of PC-AKI.

| **Variables** | | | **PC-AKI** | | | | | |  |
| --- | --- | --- | --- | --- | --- | --- | --- | --- | --- |
|  |  |  | **OR value** | | **95% CI** | | **P value** | | |
| RAR，per1-unit increase | | | 1.09 | | 1.04~1.14 | | <.001 | | |
| Age | | | 1.04 | | 1.02~1.05 | | <.001 | | |
| Female | | | 0.71 | | 0.51~1.00 | | 0.049 | | |
| Heart failure | | | 1.85 | | 1.42~2.41 | | <.001 | | |
| Smoke | | | 0.89 | | 0.67~1.18 | | 0.420 | | |
| Hypertension | | | 1.18 | | 0.91~1.54 | | 0.219 | | |
| COPD | | | 0.75 | | 0.37~1.53 | | 0.433 | | |
| Previous MI | | | 0.71 | | 0.50~1.00 | | 0.051 | | |
| Prior PCI | | | 0.94 | | 0.64~1.37 | | 0.736 | | |
| Prior Stroke | | | 1.40 | | 0.95~2.06 | | 0.088 | | |
| Anemia | | | 1.32 | | 1.01~1.72 | | 0.039 | | |
| eGFR | | | 0.99 | | 0.99~1.00 | | 0.004 | | |
| Aspirin | 0.43 | | 0.19~1.01 | | 0.052 | |  |  |  |
| GP IIb / IIIa inhibitor | | | 1.14 | | 0.87~1.48 | | 0.343 | | |
| Multi-vessel stenosis | | | 1.34 | | 1.01~1.78 | | 0.040 | | |
| Femoral access | | | 1.47 | | 1.08~1.99 | | 0.014 | | |

**Abbreviation:** PC-AKI: post-contrast acute kidney injury; RAR: random glucose to albumin ratio; COPD: chronic obstructive pulmonary disease; MI: myocardial infarction; PCI: percutaneous coronary intervention; eGFR: estimated glomerular filtration rate; GP IIb / IIIa inhibitor: Glycoprotein IIb/IIIa inhibitor.

**Supplementary Table 3.** Multivariable logistic regression analysis for the RAR as categorical variable in other definition of PC-AKI.

| **Variables** | **PC-AKI** | | |
| --- | --- | --- | --- |
|  | **OR value** | **95% CI** | **P value** |
| T1 |  | Reference |  |
| T2 | 1.24 | 0.87~1.76 | 0.239 |
| T 3 | 1.60 | 1.14~2.26 | 0.007 |
| Age | 1.03 | 1.02~1.05 | <.001 |
| Female | 0.74 | 0.53~1.03 | 0.077 |
| Heart failure | 1.88 | 1.44~2.44 | <.001 |
| Smoke | 0.89 | 0.67~1.19 | 0.436 |
| Hypertension | 1.18 | 0.91~1.55 | 0.216 |
| COPD | 0.75 | 0.37~1.53 | 0.431 |
| Previous MI | 0.70 | 0.49~0.99 | 0.045 |
| Previous PCI | 0.92 | 0.63~1.35 | 0.686 |
| Previous Stroke | 1.41 | 0.96~2.07 | 0.082 |
| Anemia | 1.32 | 1.01~1.72 | 0.039 |
| eGFR | 0.99 | 0.99~1.00 | 0.001 |
| Aspirin | 0.42 | 0.18~0.97 | 0.043 |
| GP IIb / IIIa inhibitor | 1.14 | 0.87~1.48 | 0.340 |
| Multi-vessel stenosis | 1.33 | 1.00~1.76 | 0.046 |
| Femoral access | 1.46 | 1.08~1.98 | 0.015 |

**Abbreviation:** PC-AKI: post-contrast acute kidney injury; RAR: random glucose to albumin ratio; COPD: chronic obstructive pulmonary disease; MI: myocardial infarction; PCI: percutaneous coronary intervention; eGFR: estimated glomerular filtration rate; GP IIb / IIIa inhibitor: Glycoprotein IIb/IIIa inhibitor.

**Supplementary Table 4.** Multivariable logistic regression analysis of RAR for the clinical outcomes.

| **Variables** | **PC-AKI** | | |  | **MACE** |  |
| --- | --- | --- | --- | --- | --- | --- |
|  | **OR value** | **95% CI** | **P value** | **OR value** | **95% CI** | **P value** |
| **RAR (as continuous)** |  |  |  |  |  |  |
| Model 1 | 1.11 | 1.05~1.18 | <.001 | 1.14 | 1.08~1.21 | <.001 |
| Model 2 | 1.09 | 1.02~1.15 | 0.005 | 1.11 | 1.04~1.18 | 0.001 |
| **RAR (as categorical)** |  |  |  |  |  |  |
| Model 1 |  |  |  |  |  |  |
| T1 |  | Reference |  |  | Reference |  |
| T2 | 0.99 | 0.61~1.60 | 0.972 | 1.00 | 0.61~1.66 | 0.994 |
| T3 | 1.80 | 1.11~2.92 | 0.016 | 2.47 | 1.50~4.06 | <.001 |
| Model 2 |  |  |  |  |  |  |
| T1 |  | Reference |  |  | Reference |  |
| T2 | 1.21 | 0.75~1.94 | 0.440 | 1.06 | 0.65~1.74 | 0.815 |
| T3 | 1.89 | 1.17~3.05 | 0.009 | 2.17 | 1.33~3.55 | 0.002 |

Model 1: adjusted the variables of diabetes, age, anemia, diabetes, hypertension, heart failure, GP IIb/ IIIa inhibitor, previous MI, and multi-vessel stenosis.

Model 2: adjusted the variables of diabetes, female, multi-vessel stenosis, femoral access, smoke previous stroke, eGFR, COPD, and aspirin.

**Abbreviation:** PC-AKI: post-contrast acute kidney injury; MACE: major adverse clinical events; RAR: random glucose to albumin ratio; GP IIb / IIIa inhibitor: Glycoprotein IIb/IIIa inhibitor; MI: myocardial infarction; eGFR: estimated glomerular filtration rate; COPD: chronic obstructive pulmonary disease.

**Supplementary Table 5.** Multivariable logistic regression analysis for the RAR predict the incidence of PC-AKI excluding those variables.

| RAR，per1-unit increase | **PC-AKI** | | |
| --- | --- | --- | --- |
|  | **OR value** | **95% CI** | **P value** |
| All variables in | 1.099 | 1.043~1.158 | 0.000 |
| Leave Age out | 1.088 | 1.033 1.147 | 0.002 |
| Leave Female out | 1.085 | 1.030 1.142 | 0.002 |
| Leave Heart failure out | 1.113 | 1.058 1.172 | 0.000 |
| Leave Smoke out | 1.099 | 1.043 1.158 | 0.000 |
| Leave Hypertension out | 1.099 | 1.043 1.158 | 0.000 |
| Leave COPD out | 1.101 | 1.045 1.160 | 0.000 |
| Leave Previous MI out | 1.103 | 1.047 1.161 | 0.000 |
| Leave Prior PCI out | 1.100 | 1.044 1.159 | 0.000 |
| Leave Previous Stroke out | 1.102 | 1.046 1.160 | 0.000 |
| Leave Anemia out | 1.100 | 1.044 1.159 | 0.000 |
| Leave eGFR out | 1.138 | 1.082 1.197 | 0.000 |
| Leave Aspirin out | 1.099 | 1.043 1.158 | 0.000 |
| Leave GP IIb/IIIa inhibitor out | 1.098 | 1.042 1.157 | 0.000 |
| Leave Multi-vessel stenosis out | 1.103 | 1.048 1.162 | 0.000 |
| Leave Femoral access out | 1.100 | 1.044 1.159 | 0.000 |

**Abbreviation:** PC-AKI: post-contrast acute kidney injury; RAR: random glucose to albumin ratio; COPD: chronic obstructive pulmonary disease; MI: myocardial infarction; PCI: percutaneous coronary intervention; eGFR: estimated glomerular filtration rate; GP IIb / IIIa inhibitor: Glycoprotein IIb/IIIa inhibitor.

**Supplementary Table 6.** Subgroup analysis of RAR for the clinical outcomes.

| **Subgroup** | **OR** | **95%CI** | **P value** | **Interaction**  **P value** |
| --- | --- | --- | --- | --- |
| PC-AKI |  |  |  |  |
| DM |  |  |  | 0.554 |
| With DM | 1.07 | 1.00~1.14 | 0.069 |  |
| Without DM | 1.13 | 0.99~1.30 | 0.067 |  |
| Gender |  |  |  | 0.533 |
| Male | 1.09 | 1.01~1.18 | 0.019 |  |
| Female | 1.05 | 0.94~1.18 | 0.357 |  |
| Age |  |  |  | 0.000 |
| Age ≥ 65 years | 1.11 | 1.03~1.20 | 0.007 |  |
| Age < 65 years | 1.00 | 0.91~1.11 | 0.923 |  |
| Hypertension |  |  |  | 0.409 |
| With Hypertension | 1.12 | 1.05~1.19 | 0.001 |  |
| Without Hypertension | 1.06 | 0.96~1.17 | 0.231 |  |
| MACE |  |  |  |  |
| DM |  |  |  | 0.026 |
| With DM | 1.05 | 0.97~1.13 | 0.204 |  |
| Without DM | 1.23 | 1.08~1.40 | 0.002 |  |
| Gender |  |  |  | 0.963 |
| Male | 1.11 | 1.02~1.21 | 0.012 |  |
| Female | 1.05 | 0.94~1.17 | 0.394 |  |
| Age |  |  |  | 0.354 |
| Age ≥ 65 years | 1.10 | 1.01~1.19 | 0.038 |  |
| Age< 65 years | 1.11 | 1.01~1.23 | 0.037 |  |
| Hypertension |  |  |  | 0.665 |
| With Hypertension | 1.07 | 1.00~1.15 | 0.047 |  |
| Without Hypertension | 1.08 | 0.98~1.21 | 0.134 |  |

**Abbreviation:** PC-AKI: post-contrast acute kidney injury; MACE: major adverse clinical events; DM: diabetes mellitus.

**Supplementary Figure 1**. ROC curve analysis of RAR for predicting the incidence of other definition of PC-AKI.

**
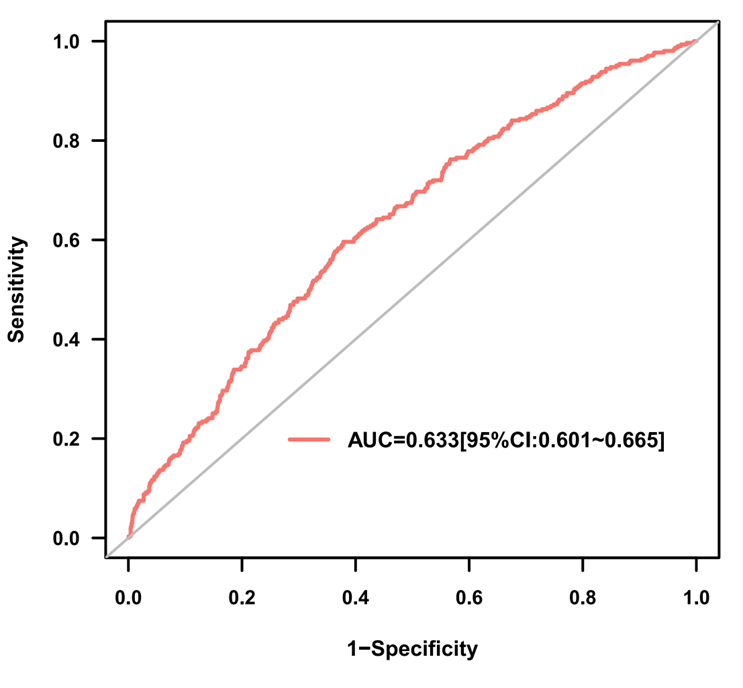
**

**Supplementary Figure 2.** Comparison of RAR predicting the incidence of PC-AKI between male and female**.**


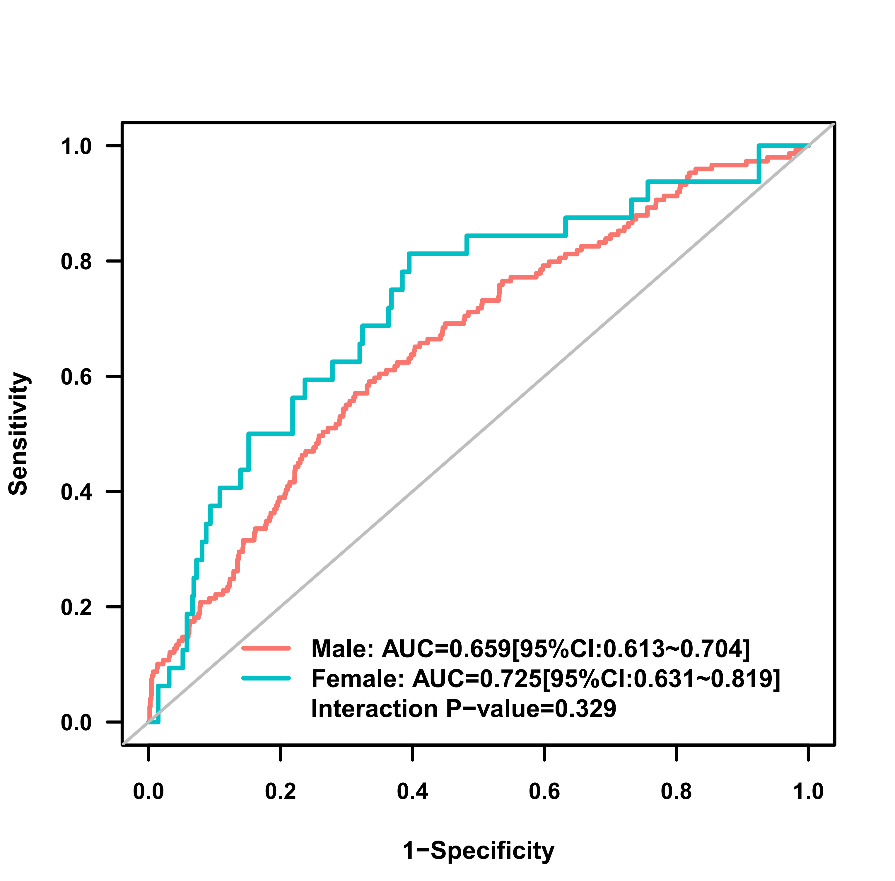

Supplement: Supplementary file 1 [file DataSheet_1.docx]
